# Sustained improvement in renal function with palopegteriparatide in adults with chronic hypoparathyroidism: 2-year results from the phase 3 PaTHway-trial

**DOI:** 10.1093/jbmr/zjag085

**Published:** 2026-05-21

**Authors:** Lars Rejnmark, Elvira O Gosmanova, Aliya A Khan, Stuart Sprague, Dolores M Shoback, Lynn Kohlmeier, Mishaela R Rubin, Andrea Palermo, Peter Schwarz, Claudia Gagnon, Elena Tsourdi, Yasuhiro Takeuchi, Noriko Makita, Yasuo Imanishi, Neil Gittoes, Juan J Díez, Caroline Prot-Bertoye, Bryant Lai, Carol Zhao, Jenny Ukena, Michael A Makara, Christopher T Sibley, Aimee D Shu

**Affiliations:** Department of Clinical Medicine and Endocrinology, Aarhus University Hospital, Aarhus, 8200 Aarhus N, Denmark; Nephrology Section, Department of Medicine, Albany Stratton VA Medical Center, Albany, NY 12008, United States; Division of Nephrology and Hypertension, Department of Medicine, Albany Medical College, Albany, NY 12208, United States; Divisions Endocrinology and Metabolism and Geriatrics, Department of Medicine, McMaster University, Hamilton, ON, L8S 4L8, Canada; Division of Nephrology and Hypertension, NorthShore University Health System, Evanston, IL 60201, United States; Department of Medicine, Pritzker School of Medicine, University of Chicago, Chicago, IL 60607, United States; Endocrine Research Unit, University of California San Francisco Medical Center, San Francisco, CA 94143, United States; Department of Internal Medicine, San Francisco VA Medical Center, San Francisco, CA 94121, United States; Spokane Osteoporosis and Endocrinology, Arthritis Northwest Research Center, Spokane, WA 99223, United States; Department of Endocrinology, Columbia University, New York, NY 10032, United States; Endocrinology and Diabetes Unit, Campus Bio-Medico University of Rome, Roma, 00128, Italy; Department of Endocrinology, Rigshospitalet, Copenhagen, 2100, Denmark; Faculté de médecine, Université Laval, Quebec City, QC, G1V 0A6, Canada; Centre de recherche du CHU de Québec-Université Laval, Quebec, QC, G1V 4G2, Canada; Department of Medicine III and Center for Healthy Aging, Dresden University of Technology, Dresden, 01307, Germany; Endocrine Center, Toranomon Hospital Kajigaya, Kawasaki, 213-8587, Japan; Okinaka Memorial Institute for Medical Research, Minato-Ku, Tokyo, 105-8470, Japan; Division of Nephrology and Endocrinology, The University of Tokyo Hospital, Tokyo, 113-8655, Japan; Department of Clinical Medical Science, Osaka Metropolitan University Graduate School of Medicine, Osaka, 545-8585, Japan; Centre for Endocrinology, Diabetes and Metabolism, Queen Elizabeth Hospital Birmingham, Birmingham, B15 2GW, United Kingdom; Department of Endocrinology, Hospital Universitario Puerta de Hierro Majadahonda, Majadahonda, 28220, Spain; Service de Physiologie-Explorations Fonctionnelles, Hôpital Européen Georges Pompidou, Paris, 75015, France; Centre de Référence des Maladies Rares du Calcium et du Phosphate, Assistance Publique Hôpitaux de Paris, Paris, 75015, France; Université Paris Cité, Paris, 75006, France; Ascendis Pharma, LLC, Palo Alto, CA 94304, United States; Ascendis Pharma, LLC, Palo Alto, CA 94304, United States; Ascendis Pharma, LLC, Palo Alto, CA 94304, United States; Ascendis Pharma, LLC, Palo Alto, CA 94304, United States; Ascendis Pharma, LLC, Palo Alto, CA 94304, United States; Ascendis Pharma, LLC, Palo Alto, CA 94304, United States

**Keywords:** clinical trials, PTH/Vit D/FGF23, parathyroid-related disorders, hormone replacement/receptor modulators, systems biology - bone interactors

## Abstract

The PaTHway clinical trial demonstrated sustained efficacy, safety, and tolerability of daily palopegteriparatide in the treatment of adults with hypoparathyroidism. Palopegteriparatide treatment was also associated with improvements in estimated glomerular filtration rate (eGFR) over 52 wk in a post hoc analysis, prompting further exploration of palopegteriparatide in the preservation of renal function. The current post hoc analysis evaluated the impact of palopegteriparatide treatment on renal function in adults with hypoparathyroidism through Week 104; as with prior analysis, changes in renal function were assessed using eGFR. At Week 104, 93% (76/82) of participants remained in the open-label extension. Of those, 82% had albumin-adjusted serum calcium levels in the normal range (8.3-10.6 mg/dL), 97% were independent from conventional therapy (≤600 mg/d of elemental calcium and no active vitamin D). Mean (SD) serum phosphate (3.5 (0.7) mg/dL) and albumin-adjusted calcium × phosphate product (30.7(5.1) mg^2^/dL^2^) levels were also within normal ranges. Palopegteriparatide treatment resulted in a mean (SD) increase in eGFR of 8.9 (11.0) mL/min/1.73m^2^ (*p <* .0001) from baseline to Week 52, which was sustained through Week 104, where it was 77.8 (14.8) mL/min/1.73 m^2^ with a mean (SD) change from baseline of 9.0 (10.3) mL/min/1.73 m^2^ (*p* < .0001). By Week 104, 45% and 34% of participants had an increase in eGFR of ≥5 mL/min/1.73 m^2^ and ≥10 mL/min/1.73 m^2^, respectively. Palopegteriparatide treatment normalized mean (SD) 24-h urine calcium within 26 wk and maintained levels below 250 mg/d through Week 104 (158.8 (90.5) mg/24 h). Most treatment-emergent adverse events were mild or moderate; no new safety signals or cases of treatment-related nephrolithiasis were reported. These findings demonstrate sustained renal safety of palopegteriparatide and suggest that PTH replacement therapy with palopegteriparatide preserves and may improve renal function in adults with chronic hypoparathyroidism after 104 wk of treatment.

## Introduction

PTH is a critical regulator of calcium and phosphate homeostasis. In the kidney, binding of PTH to PTH receptors (PTHRs) increases renal calcium reabsorption, phosphate excretion, and the conversion of 25OHD into active 1,25(OH)_2_D_3_. In patients with hypoparathyroidism, PTH replacement therapy normalizes phosphate and calcium balance and may improve renal function, even in those with baseline renal impairment.^1^ While the mechanism for this is not established, accumulating evidence indicates PTH may play a role in modulating glomerular filtration, underscoring the importance of PTH in the kidney.^2^^,^^3^ A rapid influence of PTH on GFR has been observed clinically, with a single intravenous infusion of PTH (1-34) associated with an acute 10% increase in GFR in both healthy and hypertensive patients.^4^

For patients with chronic hypoparathyroidism managed with conventional therapy (active vitamin D and calcium), renal dysfunction is common whether the disease is congenital in nature or acquired from surgical damage or removal of parathyroid glands. Compared with age- and sex-matched controls, patients with chronic hypoparathyroidism on conventional therapy are at increased risk for renal calcifications (eg, nephrocalcinosis and nephrolithiasis), substantial declines in estimated glomerular filtration rate (eGFR), CKD, and end stage kidney disease (ESKD).^5^^,^^6^ The pathophysiological mechanisms for renal complications in chronic hypoparathyroidism are not well established and likely reflect a variety of factors, including intrinsic features of hypoparathyroidism and potential adverse renal responses (eg, tubular damage) to conventional therapy.^7–9^ Preventing renal functional compromise in chronic hypoparathyroidism requires therapies that not only obviate the need for conventional therapy but also restore physiologic PTH1R activation in the kidneys and restore physiologic levels of PTHR signaling. While individuals with chronic hypoparathyroidism receiving daily rhPTH (1-84) therapy have shown preservation of renal function vs historical controls,^10^ administration of short-lived PTH therapy leads to transient, supraphysiologic spikes in exogenous PTH followed by significant periods of inadequate serum PTH concentration and continued requirement for conventional therapy.^3^

Palopegteriparatide is a prodrug of PTH (1-34), administered subcutaneously once daily, designed to provide active PTH within the physiological range for 24 h/d in adults with hypoparathyroidism.^11^ It is approved in the United States (US), European Union (EU) and European Economic Area, Australia, Japan, and several other regions.^12–17^ The Phase 3 PaTHway trial in adults with chronic hypoparathyroidism showed that significantly more participants treated with palopegteriparatide (*n* = 48/61, 79%) met the primary multi-component efficacy endpoint at Week 26, defined as normocalcemia plus independence from conventional therapy (≤600 mg/d of elemental calcium and no active vitamin D) with no increase in study drug during the 4 wk before Week 26, compared with those receiving placebo (*n* = 1/21, 5%) (*p* < .0001).^18^ Participants receiving palopegteriparatide also demonstrated significant improvements in multiple hypoparathyroidism-specific measures evaluating physical and cognitive symptoms and the impact of hypoparathyroidism on physical functioning and daily life.^18^ Mean 24-h urine calcium levels normalized by Week 26 in participants receiving palopegteriparatide, while mean values remained elevated in those allocated to placebo. Serum phosphate remained in the normal range in both arms.

Interestingly, post hoc analyses from the PaTHway trial showed that palopegteriparatide treatment was also associated with clinically significant improvements in eGFR at Week 26 that were sustained through Week 52.^1^ Participants receiving palopegteriparatide had a mean (SD) increase in eGFR of 9.3 (11.7) mL/min/1.73 m^2^ over 52 wk, and 43% had an increase of ≥10 mL/min/1.73 m^2^.^1^ Participants with baseline eGFR <60 mL/min/1.73 m^2^ had a mean (SD) increase of 11.5 (11.3) mL/min/1.73 m^2^ over the first 52 wk of treatment with palopegteriparatide.^1^ Previous reports of patients with hypoparathyroidism managed with conventional therapy have described annualized declines in eGFR ranging from −0.7 to −1.8 mL/min/1.73m^2^/yr.^3^^,^^10^^,^^19^^,^^20^ The current post hoc analyses describe longitudinal changes in renal function over the first 2 yr of the PaTHway trial, demonstrating sustained increases in eGFR for the total study population and subgroups with baseline eGFR <60 and those with baseline eGFR ≥60 mL/min/1.73 m^2^.

## Materials and methods

### Trial design and participants

The Phase 3 PaTHway trial was a randomized, double-blind trial comprising a 26-wk placebo-controlled treatment period followed by a 156-wk open label extension (ClinicalTrials.gov ID: NCT04701203; EudraCT ID: 2020-003380-26). PaTHway was designed to evaluate the efficacy, safety, and tolerability of palopegteriparatide in adults with chronic hypoparathyroidism with the design described previously.^18^ Briefly, men and women with chronic hypoparathyroidism were enrolled, including those with postsurgical, genetic, autoimmune, and idiopathic etiologies. Trial participants were required to have an eGFR of ≥30 mL/min/1.73 m^2^ and urinary calcium excretion ≥125 mg/24 h at screening. Use of loop diuretics and phosphate binders (apart from oral calcium) were not allowed during the blinded period, and thiazide diuretic use was not permitted within 4 wk prior to the 24-h urine collection before visit 1 or during the blinded period.

Enrolled participants were randomized 3:1 to receive palopegteriparatide by once-daily s.c. injection at an initial dose of 18 μg PTH (1-34)/d or a corresponding volume of placebo, both of which were co-administered with conventional therapy consisting of active vitamin D and elemental calcium. Doses of palopegteriparatide (or corresponding placebo) and conventional therapy were titrated according to a protocol-specified algorithm guided by serum calcium levels.^18^ After Week 26, participants randomized to placebo during the blinded period were eligible to begin palopegteriparatide treatment at 18 μg PTH (1-34)/d while continuing their conventional therapy. These individuals were then titrated to an optimized palopegteriparatide dose, concurrent with reduction or removal of conventional therapy, using the same protocol-specified dosing algorithm.

The study protocol was reviewed by the appropriate institutional review boards and independent ethics committees. The trial was designed by the sponsor and authors and was conducted in accordance with the principles of the Declaration of Helsinki and Good Clinical Practice guidelines as described in the International Conference on Harmonization Guideline E6. The current report describes results through Week 104 of the PaTHway trial.

### Outcome measures

#### Treatment efficacy

Efficacy of palopegteriparatide during the open-label extension period was evaluated with a multi-component endpoint comprising the proportion of participants with normocalcemia (ie, albumin-adjusted serum calcium 8.3-10.6 mg/dL or 2.1-2.6 mmol/L) and independence from conventional therapy, defined as no active vitamin D intake and elemental calcium intake of ≤600 mg/d on the day prior to the visit of interest.

#### Renal function

Renal function was assessed by eGFR, derived using the Modified Diet in Renal Disease (MDRD) equation: eGFR (mL/min/1.73 m^2^) = 175 × (serum creatinine in mg/dL)^−1.154^ × age^−0.203^ × 0.742 [if female] × 1.212 [if of Black race].^21^ The use of this equation was prespecified in the PaTHway trial design. eGFR was determined at baseline and at regular intervals throughout the blinded and open-label extension periods, with outcomes of interest including mean eGFR at prespecified time points and changes in eGFR from baseline through Week 104.

Change in eGFR for the overall population and by treatment group was also evaluated by determining eGFR slope between Weeks 0-26, Weeks 26-52, and Weeks 52-104. eGFR slope is a Committee for Medicinal Products for Human Use-qualified validated surrogate endpoint for CKD progression, with treatment-related improvement of 0.75 mL/min/1.73 m^2^ per yr predicting a clinical benefit on progression of CKD at the population level.^22^

Responder analyses were performed to evaluate the percentage of participants with a clinically meaningful change from baseline in eGFR of ≥5 mL/min/1.73 m^2^,^23^^,^^24^ as well as change from baseline of ≥10 mL/min/1.73 m^2^, at Weeks 26, 52, and 104.

All renal outcomes were analyzed for the overall trial population and stratified by baseline eGFR (<60 and ≥60 mL/min/1.73 m^2^).

#### Safety

Safety assessments related to treatment included 24-h urine calcium excretion obtained at prespecified time points during the blinded and open-label extension periods, as well as treatment-emergent adverse events (TEAEs). The severity, seriousness, and causality of all reported TEAEs were evaluated by the trial site investigators. Adverse events (AEs) were coded by system organ class (SOC) and preferred term (PT) using the Medical Dictionary for Regulatory Activities (MedDRA).

#### Statistical analyses

Data were summarized descriptively by treatment group and the overall population with continuous variables presented as mean and SD or median and range, with categorical data presented using counts (*n*) and percentages of participants. Baseline was defined as the last assessment prior to the first dose of the assigned treatment during the blinded period. At post-baseline visits, only data from participants with both baseline and the corresponding visit values were used to compute statistical summaries. Data were summarized based on treatment assignment in the blinded period as well as for all participants combined, aligned to their first dose of palopegteriparatide received. Paired *t* tests were used to examine if changes from baseline in eGFR in individual treatment groups were greater than zero. eGFR slope and 95% confidence intervals were estimated using a mixed model repeated measures (MMRM) model with change in eGFR as the dependent variable, baseline eGFR and yr of palopegteriparatide treatment as covariates, and participant as a random effect. Correlation between change in eGFR and change in conventional therapy doses were assessed using Pearson correlation. Subgroup analysis was performed in participants with baseline eGFR <60 and ≥60 mL/min/1.73 m^2^. Adverse events were summarized between the first dose of palopegteriparatide and date of data extraction for analysis on July 26, 2023. For participants randomized to placebo at enrollment, only AEs occurring during the open-label period on palopegteriparatide were included. All statistical analyses were conducted using SAS version 9.4, and data were analyzed as previously described.^1^

## Results

### Participant demographics and baseline characteristics

Eighty-two participants enrolled in the PaTHway trial were randomized and received at least one dose of blinded study drug (*n* = 61 palopegteriparatide, *n* = 21 placebo), 79 (96%) of whom completed blinded treatment through Week 26 and 76 (93%) of whom completed Week 104. Sixty-four participants (78%) were female and 76 (93%) were White. The overall mean baseline age (SD) was 48.6 (12.7) yr, and mean duration of hypoparathyroidism at baseline (SD) was 11.7 (10.7) yr. Eighty-five percent of participants (70/82) had acquired hypoparathyroidism from neck surgery (Table 1), and groups were comparable for baseline prevalence of hypertension and diabetes and the use of medications that may affect eGFR, such as nonsteroidal anti-inflammatory drugs, thiazide and other diuretics, angiotensin-converting enzyme inhibitors, angiotensin receptor blockers, GLP-1 agonists, sodium-glucose cotransporter 2 inhibitors, proton pump inhibitors, and histamine 2 (H2) receptor antagonists.

**Table 1:** Demographics and baseline clinical characteristics of PaTHway trial participants (overall and by baseline renal function).

	**All participants (*n* = 82)**	**Baseline eGFR** **<60 mL/min/1.73 m** ^**2**^ **(*n* = 23)**	**Baseline eGFR** **≥60 mL/min/1.73 m** ^**2**^ **(*n* = 59)**
**Age (yr), mean (SD)**	48.6 (12.7)	53.9 (12.7)	46.5 (12.1)
**Female, *n* (%)**	64 (78.0)	18 (78.3)	46 (78.0)
**Race, *n* (%)**			
**White**	76 (92.7)	21 (91.3)	55 (93.2)
**Asian**	5 (6.1)	1 (4.3)	4 (6.8)
**Other**	1 (1.2)	1 (4.3)	0 (0.0)
**Weight in kg, mean (SD)**	78.3 (16.9)	80.5 (15.7)	77.5 (17.5)
**Body mass index in kg/m** ^**2**^ **, mean (SD)**	27.8 (5.9)	29.4 (5.6)	27.2 (5.9)
**Hypoparathyroidism acquired from neck surgery, *n* (%)**	70 (85.4)	18 (78.3)	52 (88.1)
**Duration of hypoparathyroidism in yr, mean (SD)**	11.7 (10.7)	17.4 (14.5)	9.5 (7.8)
**Total daily dose of conventional therapy at baseline, mean (SD)**			
**Elemental calcium (mg)**	1839.4 (1049.6)	2207.6 (1550.8)	1695.8 (742.7)
***N***	82	23	59
**Calcitriol (μg)**	0.75 (0.3)	0.9 (0.4)	0.7 (0.3)
***N***	70	19	51
**Alfacalcidol (μg)**	2.3 (0.8)	2.1 (0.6)	2.4 (0.9)
***N***	12	4	8
**Medical history, *n* (%)**			
**Hypertension**	20 (24.4)	7 (30.4)	13 (22.0)
**Type 2 diabetes**	3 (3.7)	1 (4.3)	2 (3.4)
**Nephrolithiasis**	18 (22.0)	5 (21.7)	13 (22.0)
**Renal insufficiency**^**a**^	6 (7.3)	6 (26.1)	0
**Prior medication use at baseline, *n* (%)**			
**NSAIDs**^**b**^	12 (14.6)	5 (21.7)	7 (11.9)
**Diuretics, all forms**	15 (18.3)	4 (17.4)	11 (18.6)
**Thiazide diuretics**^**c**^	11 (13.4)	4 (17.4)	7 (11.9)
**ACE inhibitors**	7 (8.5)	3 (13.0)	4 (6.8)
**ARBs**	6 (7.3)	2 (8.7)	4 (6.8)
**Proton pump inhibitors**	13 (15.9)	4 (17.4)	9 (15.3)
**H2-receptor antagonists**	1 (1.2)	0	1 (1.7)
**GLP-1 agonists**	1 (1.2)	0	1 (1.7)
**SGLT2 inhibitors**	0	0	0
**Laboratory values, mean (SD)** ^**d**^			
**Serum**			
**Calcium, albumin-adjusted (mg/dL)**	8.7 (0.7)	8.9 (0.8)	8.7 (0.6)
**Phosphate (mg/dL)**	4.1 (0.7)	4.1 (0.6)	4.2 (0.7)
**Calcium × phosphate product (mg**^**2**^**/dL**^**2**^**)**	35.6 (6.3)	36.3 (5.2)	36.1 (6.5)
**Creatinine (mg/dL)**	1.0 (0.2)	1.2 (0.2)	0.9 (0.1)
** eGFR** ^**e**^ **(mL/min/1.73 m**^**2**^**)**	68.3 (13.3)	52.1 (5.6)	75.2 (10.6)
**Urine**			
**24-hour urine calcium (mg/d)**	375.6 (168.4)	376.2 (176.1)	375.4 (166.8)
**24-hour urine creatinine (mg/d)**	1273.7 (397.1)	1264.5 (431.4)	1277.3 (386.9)
**24-hour urine phosphate (g/d)**	0.668 (0.3)	0.697 (0.3)	0.655 (0.3)

a Clinician-reported history of renal insufficiency.

b Baseline NSAID use included propionic acid derivatives, acetic acid derivatives, oxicams, and salicylic acid. Ten participants reported oral NSAID use, and 3 participants reported topical NSAID use (1 participant reported both topical and oral NSAID use).

c All participants were required to discontinue thiazide diuretics at least 4 wk prior to visit 1, per protocol.

d Reference ranges: Serum calcium, albumin-adjusted: 8.3-10.6 mg/dL; serum phosphate: 3.0-4.3 mg/dL for females and 2.8–4.1 ng/dL for males; serum calcium × phosphate product: <55 mg^2^/dL^2^; serum creatinine: 0.57-1.00 mg/dL for females and 0.76-1.27 mg/dL for males; eGFR: >90 mL/min/.73 m^2^; 24-h urine calcium, ≤250 mg/d for females and ≤300 mg/d for males; 24-h urine creatinine: 400-1600 mg/d for females and 1000-2500 mg/d for males; 24-h urine phosphate: 0.26-1.08 g/d for females and 0.39-1.42 g/d for males.

e eGFR derived using the 4-variable, Modification in Diet in Renal Disease (MDRD) equation.^21^

ACE, angiotensin-converting enzyme; ARB, angiotensin receptor blocker; eGFR, estimated glomerular filtration rate; NSAID, nonsteroidal anti-inflammatory drug; SGLT2, sodium-glucose cotransporter 2.

Mean (SD) baseline eGFR levels were 67.3 (13.7) and 72.7 (14.6) mL/min/1.73 m^2^ for those randomized to palopegteriparatide and placebo, respectively. At baseline, 23 of 82 participants (28%) had eGFR <60 mL/min/1.73 m^2^, including 19 randomized to palopegteriparatide and 4 randomized to placebo. The remaining 72% of participants (59/82) had baseline eGFR ≥60 mL/min/1.73 m^2^, including 42 who received palopegteriparatide and 17 who received placebo Table 1). Participants with baseline eGFR <60 mL/min/1.73 m^2^ had a greater mean age (53.9 vs 46.5 yr), with higher mean baseline daily doses of elemental calcium (2207.6 mg vs 1695.8 mg) and calcitriol (0.9 μg vs 0.7 μg), longer mean duration of hypoparathyroidism (17.4 vs 9.5 yr), and a higher proportion of non-surgical etiologies for their disease vs those with baseline eGFR ≥60 mL/min/1.73 m^2^. All 6 participants who reported a history of renal insufficiency had a baseline eGFR <60 mL/min/1.73 m^2^.

### Treatment efficacy

Palopegteriparatide treatment during the blinded period enabled rapid and sustained reduction of elemental calcium doses to ≤600 mg/d and complete discontinuation of active vitamin D use within 8 wk, as described previously.^18^ Of 76 participants treated with palopegteriparatide through Week 104 of the PaTHway trial, 61 (80%) met the multi-component efficacy endpoint, with all 76 participants (100%) achieving independence from active vitamin D, 74 (97%) achieving independence from therapeutic doses of calcium (≤600 mg/d), and 62 (82%) maintaining albumin-adjusted serum calcium within the normal range (Table 2). Across the baseline eGFR subgroups, comparable efficacy response rates were observed through Week 104, with effectively similar rates of independence from active vitamin D and therapeutic doses of elemental calcium and maintenance of normocalcemia.

**Table 2:** PaTHway trial multi-component efficacy outcome at Week 104 (overall and by baseline renal function).

	**All participants** **(*n* = 82)**	**Baseline eGFR** **<60 mL/min/1.73 m** ^**2**^ **(*n* = 23)**	**Baseline eGFR** **≥60 mL/min/1.73 m** ^**2**^ **(*n* = 59)**
**Number (%) of participants with data on all efficacy components at Week 104**	76 (92.7)	22 (95.7)	54 (91.5)
**Number of participants meeting efficacy endpoint at Week 104 (responders)** ^**a**^	61	17	44
**Percentage (95% CI)**	80.3 (69.5, 88.5)	77.3 (54.6, 92.2)	81.5 (68.6, 90.7)
**Number (%) of participants meeting each component:**			
**Albumin-adjusted serum calcium within normal range**^**b**^	62 (81.6)	17 (77.3)	45 (83.3)
**Independence from active vitamin D**	76 (100)	22 (100)	54 (100)
**Independence from therapeutic doses of calcium**	74 (97.4)	21 (95.4)	53 (98.1)

a Defined as a standing dose of active vitamin D equal to 0 and elemental calcium ≤600 mg on the day prior to the Week 104 visit.

b Normal range for albumin-adjusted serum calcium is 8.3-10.6 mg/dL (2.07-2.64 mmol/L).

Abbreviations: CI, confidence interval; eGFR, estimated glomerular filtration rate.

### Renal function

#### eGFR changes

**Figure 1:**
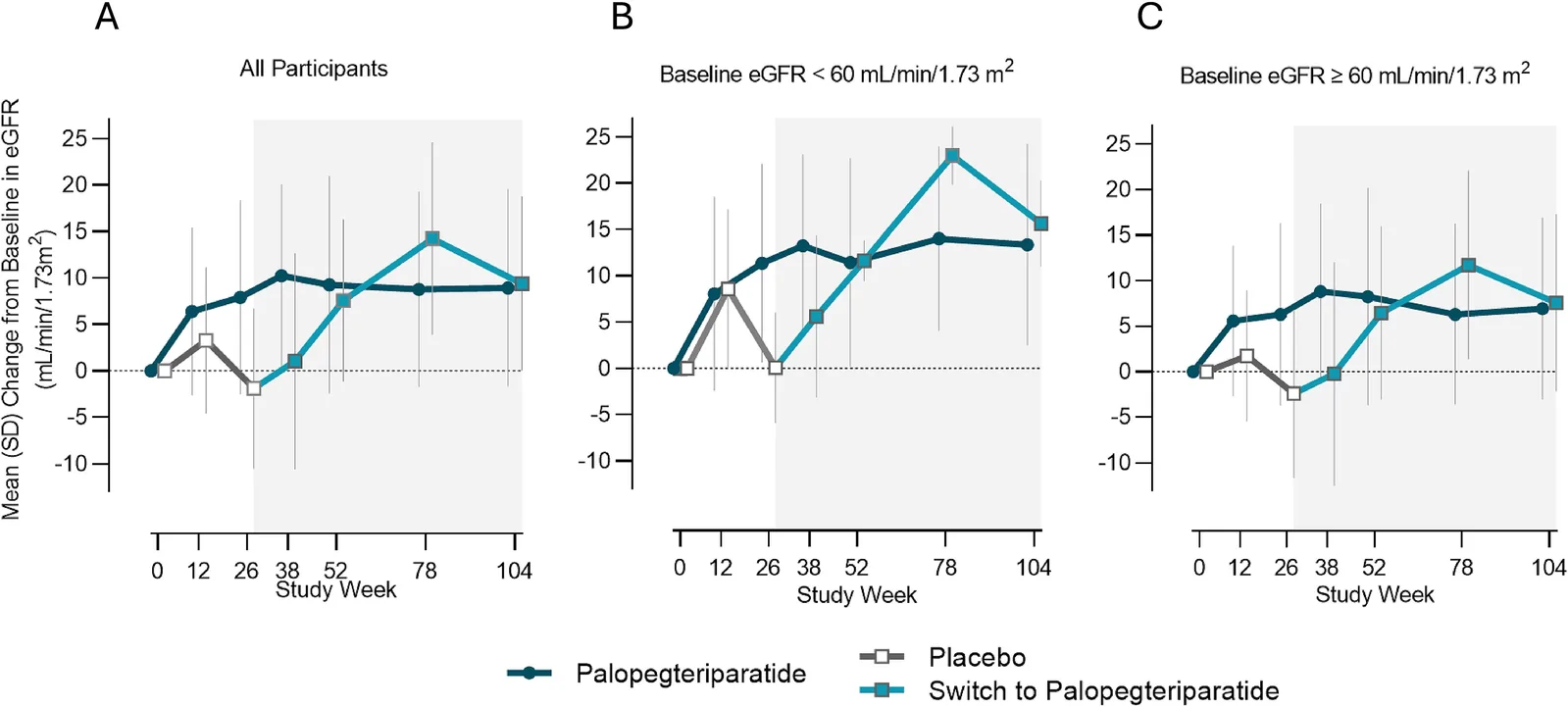
Change from baseline in eGFR through Week 104. Mean (SD) changes from baseline in estimated glomerular filtration rate (eGFR) through Week 104 for the overall population (panel A) and by baseline eGFR sub-group (<60 [panel B] or ≥60 mL/min/1.73 m^2^ [panel C]). Participants randomized to palopegteriparatide at baseline (*n* = 61) are shown in dark blue lines and symbols, and participants randomized to placebo (*n* = 21) are shown in gray lines and white symbols during the blinded period and light blue lines and symbols starting at Week 26 when their palopegteriparatide treatment was initiated (shaded region). At baseline, 19 participants randomized to palopegteriparatide and 4 randomized to placebo had baseline eGFR <60 mL/min/1.73 m^2^.

Palopegteriparatide treatment led to significantly increased eGFR from baseline to Week 52, which was maintained through Week 104 in the overall population and in the subgroups with baseline eGFR <60 and ≥60 mg/mL/1.73 m^2^ (all *p* < .0001) (Table 3). As reported previously, the greatest rate of increase in eGFR occurred during the first 26 wk of palopegteriparatide therapy.^1^ The slope continued to be positive through Week 52 and was reduced thereafter, with mean eGFR values stabilizing from Week 52 to Week 104 (Table 4). Change from baseline in eGFR by treatment group showed similar increases through Week 104 (Figure 1). At Week 104, mean eGFR (SD) remained significantly increased from baseline by 8.9 (10.6) mL/min/1.73 m^2^ in the palopegteriparatide group and 9.4 (9.4) mL/min/1.73 m^2^ in the placebo/palopegteriparatide group and was consistent with change in eGFR observed at Week 52 (Figure 1). Across both treatment groups, participants with baseline eGFR <60 mL/min/1.73 m^2^ had numerically greater sustained increases in eGFR at Weeks 52 and 104 compared with those with baseline eGFR ≥60 mL/min/1.73 m^2^ (Figure 1, Table S1). Changes in eGFR based on time on palopegteriparatide therapy are depicted in Figure S1 for the overall trial population and by baseline eGFR (<60 vs ≥60 mL/min/1.73 m^2^), demonstrating a consistent pattern of eGFR change following initiation of palopegteriparatide treatment. Absolute eGFR levels over time are shown in Figure S2. Through Week 104, no participants experienced a ≥30% sustained decrease in eGFR, a threshold for progression of kidney disease.^25^

**Table 3:** Change from baseline in eGFR at Week 52 and 104 with palopegteriparatide treatment in the PaTHway trial.

	**All participants**	**Baseline eGFR** **<60 mL/min/1.73 m** ^**2**^	**Baseline eGFR** **≥60 mL/min/1.73 m** ^**2**^
**Change from baseline in eGFR at Week 52 (mL/min/1.73 m** ^**2**^ **)** ^**a**^			
***N***	78	23	55
**Mean (SD)**	8.9 (11.0)	11.5 (10.2)	7.8 (11.3)
***p* value** ^**b**^	<.0001	<.0001	<.0001
			
**Change from baseline in eGFR at Week 104 (mL/min/1.73 m** ^**2**^ **)** ^**c**^			
**N**	76	22	54
**Mean (SD)**	9.0 (10.3)	13.8 (10.0)	7.1 (9.8)
***p* value** ^**b**^	<.0001	<.0001	<.0001

a All participants received palopegteriparatide from Week 26 to Week 52, reflecting 52 wk of palopegteriparatide treatment for palopegteriparatide participants and 26 wk of palopegteriparatide for placebo/palopegteriparatide participants.

b Obtained from a paired *t-*test, testing if the change is >0.

c All participants received palopegteriparatide from Week 26 to Week 104, reflecting 104 wk of palopegteriparatide treatment for palopegteriparatide participants and 78 wk of palopegteriparatide treatment for placebo/palopegteriparatide participants.

Abbreviations: eGFR, estimated glomerular filtration rate; SD, standard deviation; CI, confidence interval.

**Table 4:** Estimated glomerular filtration rate (eGFR) slope in participants treated with palopegteriparatide in the PaTHway trial.

**Study period**	**Parameter**	**Palopegteriparatide/** **palopegteriparatide**	**Placebo/** **palopegteriparatide**	***p*-value** ^**b**^ **(palopegteriparatide vs placebo)**
**Week 0-26** **(double-blind period)**	Slope, mL/min/1.73 m^2^/yr (SE)	15.67 (3.05)	−2.73 (4.52)	.0004
	95% CI	9.678, 21.669	−11.64, 6.185	
	*p* value^a^	<.0001	.5463	
**Week 26-52** **(open label extension)**	Slope, mL/min/1.73 m^2^/yr (SE)	2.73 (2.99)	15.69 (5.25)	.0857
	95% CI	−3.154, 8.608	5.231, 26.151	
	*p* value^a^	.362	.0038	
**Week 52-104** **(open label extension)**	Slope, mL/min/1.73 m^2^/yr (SE)	−0.29 (1.42)	1.96 (2.76)	.4121
	95% CI	−3.095, 2.519	−3.634, 7.554	
	*p* value^a^	.8391	.4816	
**Baseline eGFR <60 mL/min/1.73 m^2^**				
**Week 0-26** **(double-blind period)**	Slope, mL/min/1.73 m^2^/yr (SE)	19.24 (5.55)	−4.70 (9.04)	.0326
	95% CI	8.271, 30.206	−23.10, 13.694	
	*p* value^a^	.0007	.6064	
**Week 26-52** **(open label extension)**	Slope, mL/min/1.73 m^2^/yr (SE)	0.57 (4.81)	24.77 (5.30)	.1442
	95% CI	−9.005, 10.147	13.486, 36.059	
	*p* value^a^	.9057	.0003	
**Week 52-104** **(open label extension)**	Slope, mL/min/1.73 m^2^/yr (SE)	1.94 (1.76)	3.98 (2.63)	.6767
	95% CI	−1.628, 5.508	−2.236, 10.186	
	*p* value^a^	.2775	.1739	
**Baseline eGFR ≥60 mL/min/1.73 m^2^**				
**Week 0-26** **(double-blind period)**	Slope, mL/min/1.73 m^2^/yr (SE)	12.40 (3.54)	−4.65 (4.84)	.0041
	95% CI	5.427, 19.368	−14.23, 4.933	
	*p* value^a^	.0005	.3389	
**Week 26-52** **(open label extension)**	Slope, mL/min/1.73 m^2^/yr (SE)	3.64 (3.61)	13.64 (6.18)	.2235
	95% CI	−3.490, 10.773	1.257, 26.014	
	*p* value^a^	.3148	.0314	
**Week 52-104** **(open label extension)**	Slope, mL/min/1.73 m^2^/yr (SE)	−1.32 (1.86)	1.41 (3.33)	.4306
	95% CI	−5.014, 2.374	−5.429, 8.253	
	*p* value^a^	.479	.6753	

a Test if change of eGFR is equal to 0.

b *P*-value for testing difference between 2 treatment arms in slopes using mixed model repeated measures (MMRM) with change from baseline as the dependent variable, time, treatment, and their interaction as independent variables, and baseline eGFR as a covariate.

Slope for each treatment arm was estimated by a repeated measure mixed-effect model with change from baseline as the dependent variable, time as an independent variable, and baseline eGFR as a covariate. Abbreviations: CI = confidence interval; SE = standard error.

#### eGFR responder analysis

**Figure 2:**
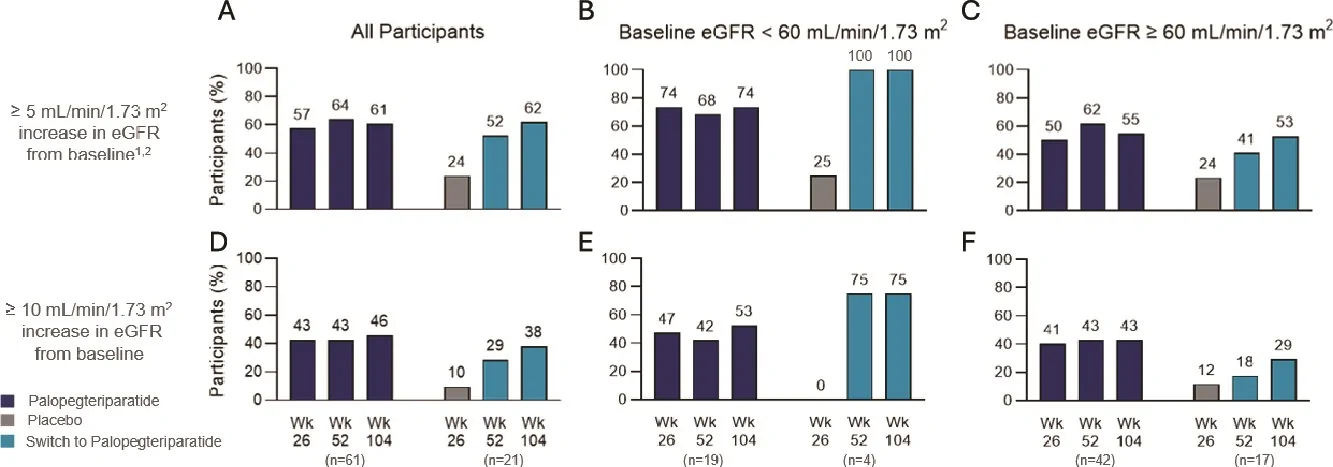
Percentage of participants with ≥5 mL and ≥10 mL/min/1.73 m^2^ in eGFR. Proportion of participants from the PaTHway trial with an increase ≥5 mL/min/1.73 m^2^ (panels A-C) and ≥ 10 mL/min/1.73 m^2^ (panels D-F) in eGFR from baseline to Weeks 26, 52, and 104. Participants randomized to palopegteriparatide at baseline (*n* = 61) are shown in dark blue, and participants randomized to placebo (*n* = 21) are shown in white during the blinded period and light blue starting at Week 26 when palopegteriparatide treatment was initiated. eGFR, estimated glomerular filtration rate.

As shown in Figure 2, 57% (35/61) of participants receiving palopegteriparatide and 23.8% (5/21) of participants receiving placebo experienced an eGFR increase of ≥5 mL/min/1.73 m^2^ through Week 26 (Figure 2, odds ratio 4.31, 95% CI = 1.398, 13.270; *p* = .0083). The likelihood of experiencing an eGFR increase of ≥5 mL/min/1.73 m^2^ at Week 26 was numerically increased for participants with an eGFR <60 mL/min/1.73 m^2^ (odds ratio 8.40, 95% CI = 0.701, 100.59; *p* = .0692) relative to participants with an eGFR ≥60 mL/min/1.73 m^2^ (odds ratio 3.25, 95% CI = 0.91, 11.61; *p* = .0647).

By Week 52, when all participants were treated with palopegteriparatide, 61.0% (50/82) of the overall population achieved an eGFR increase of ≥5 mL/min/1.73 m^2^, comprising 17 of 23 (73.9%) of participants with baseline eGFR <60 mL/min/1.73 m^2^ and 33 of 59 (55.9%) of participants with baseline eGFR ≥60 mL/min/1.73 m^2^.

Response rates were relatively unchanged through Week 104, with 61.0% (50/82) of the overall population achieving an eGFR increase of ≥5 mL/min/1.73 m^2^, including 78.3% (18/23) of participants with baseline eGFR <60 mL/min/1.73 m^2^ and 54.2% (32/59) of participants with baseline eGFR ≥60 mL/min/1.73 m^2^.

Similar trends were observed for percentages of participants achieving an increase in eGFR ≥10 mL/min/1.73 m^2^ (Figure 2). At Week 26, 42.6% (26/61) of participants in the palopegteriparatide group and 9.5% (2/21) of participants in the placebo group achieved an increase in eGFR ≥10 mL/min/1.73 m^2^ (odds ratio 7.057, 95% CI = 1.509, 33.013; *p* = .0061). At Week 52, when all participants were being treated with palopegteriparatide, the proportion achieving ≥10 mL/min/1.73 m^2^ improvement was 39.0% (32/82) for the overall population, 47.8% (11/23) for participants with baseline eGFR <60 mL/min/1.73 m^2^, and 35.6% (21/59) for participants with baseline eGFR ≥60 mL/min/1.73 m^2^. At Week 104, response rates for total participants, those with eGFR <60 mL/min/1.73 m^2^, and those with eGFR ≥60 mL/min/1.73 m^2^ were 43.9% (36/82), 56.5% (13/23), and 39.0% (23/59), respectively.

#### Correlations between eGFR and treatment parameters

Exploratory analyses indicated weak inverse correlations between change in eGFR and reduction of conventional therapy for trial participants. Through Week 104, the Pearson correlation coefficient for change in active vitamin D and change in eGFR for all participants was −0.22 (*p* < .001), while the correlation coefficient for change in elemental calcium and change in eGFR was −0.25 (*p* < .001). Participants remained independent from conventional therapy at Week 104, while maintaining serum biochemistries within the normal range.

### Serum and urine biochemistries

Albumin-adjusted serum calcium at Week 104 was largely unchanged from baseline, with reductions in serum phosphate and calcium × phosphate product at Week 104. In line with changes in eGFR, serum creatinine also decreased from baseline to Week 104. At Week 104, 24-h urine calcium was substantially reduced from baseline values (Table 1), concurrent with an increase in 24-h urine phosphate. The effects observed on serum calcium and phosphate and urine calcium excretion are in alignment with the expected effect of PTH replacement in hypoparathyroidism (Table 5).

**Table 5:** Serum and urine chemistries at Week 104 by baseline renal function in participants treated with palopegteriparatide in the PaTHway trial.

	**All Participants** **(*N* = 61)**		**Baseline eGFR** **<60 mL/min/1.73 m** ^**2**^ **(*N* = 19)**		**Baseline eGFR** **≥60 mL/min/1.73 m** ^**2**^ **(*N* = 42)**	
**Biochemical parameter**	**Mean** **(SD)**	**Change from baseline (SD); *p* value**	**Mean** **(SD)**	**Change from baseline (SD); *p* value**	**Mean** **(SD)**	**Change from baseline (SD); *p* value**
Serum^a^						
Calcium^b^ (mg/dL)	8.7 (0.5)	−0.1 (0.8); *p* = .7329	8.7 (0.5)	−0.3 (0.9); *p* = .1540	8.7 (0.6),	0.0 (0.7); *p* = .4418
Phosphate (mg/dL)	3.6 (0.6)	−0.6 (0.6); *p* < .0001	3.5 (0.7)	−0.6 (0.5); *p* = .0006	3.6 (0.6)	−0.7 (0.6); *p* < .0001
Calcium × phosphate product (mg^2^/dL^2^)^a^	31.1 (4.9)	−5.8 (6.0); *p* < .0001	30.2 (5.8)	−6.1 (5.9); *p* < .0001	31.5 (4.5)	−5.7 (6.2); *p* < .0001
Creatinine (mg/dL)	0.9 (0.2)	−0.1 (0.1); *p* < .0001	1.0 (0.3)	−0.2 (0.1); *p* < .0001	0.8 (0.1)	−0.1 (0.1); *p* < .0001
Urine						
Calcium^c^ (mg/24 h)	151.1 (90.4)	−239.1 (178.1); *p* < .0001	144.6 (76.3)	−221.9 (182.7); *p* = .0001	153.3 (95.8)	−245.0 (179.1); *p* < .0001
Phosphate^d^ (g/24 h)	0.9 (0.4)	0.2 (0.4); *p* = .0001	0.9 (0.4)	0.2 (0.3); *p* = .0642	0.9 (0.4)	0.2 (0.4); *p* = .001
Creatinine^e^ (mg/24 h)	1347.7 (548.1)	26.0 (434.4); *p* = .2274	1383.6 (499.3)	15.3 (411.5); *p* = .7559	1336.1 (570.2)	29.5 (448.1); *p* = .2365

a Fifty-eight participants with available data at Week 104 (40 with baseline eGFR ≥60 mL/min/1.73 m^2^, 18 with baseline eGFR <60 mL/min/1.73 m^2^).

b Corrected for albumin. Participants who had values at baseline and Week 104 were included in the statistical summary.

c Forty-three participants with available data at Week 104 (32 with baseline eGFR ≥60 mL/min/1.73 m^2^, 11 with baseline eGFR <60 mL/min/1.73 m^2^).

d Forty participants with available data at Week 104 (30 with baseline eGFR ≥60 mL/min/1.73 m^2^, 10 with baseline eGFR <60 mL/min/1.73 m^2^).

e Forty-one participants with available data at Week 104 (31 with baseline eGFR ≥60 mL/min/1.73 m^2^, 10 with baseline eGFR <60 mL/min/1.73 m^2^).

Serum and urine biochemistry values were similar across baseline eGFR and treatment allocation subgroups. Participants experienced significant reductions from baseline in 24-h urine calcium excretion at Week 104. Those with baseline eGFR <60 mL/min/1.73 m^2^ had a mean (SD) calcium excretion of 137.6 (68.5) mg/d at Week 104 (compared with 376.2 (176.1) mg/d at baseline, *p* = .0001), while those with baseline eGFR ≥60 mL/min/1.73 m^2^ had a mean (SD) excretion of 165.7 (96.3) mg/d at Week 104 (compared with 375.4 (166.8) mg/d at baseline, *p* < .0001).

### Safety

No new safety signals were identified from Week 52 to 104 during the open-label extension and no cases of treatment-related nephrolithiasis were reported through Week 104 of palopegteriparatide treatment. Two participants reported treatment-related serious AEs (1 case of hypercalcemia in each eGFR subgroup). Six participants experienced TEAEs related to hypercalcemia or hypocalcemia leading to ER visits/hospitalization (4 participants with baseline eGFR <60 mL/min/1.73 m^2^ and 2 participants with baseline eGFR ≥60 mL/min/1.73 m^2^ (Table 6)). In the overall population, 1 of 80 participants had a TEAE leading to discontinuation of study drug (grade 2 hypocalcemia) during the open-label extension, and 1 of 80 participants had a TEAE leading to death, the latter resulting from cardiac arrest (unrelated to study drug) during blinded treatment. The most commonly reported (occurring ≥5%) treatment-related AEs through Week 104 included injection site reactions (26.3%), hypercalcemia (13.8%), nausea (8.8%), hypocalcemia (7.5%), headache (7.5%), and postural orthostatic tachycardia syndrome (5.0%). There was no incident nephrocalcinosis through Week 104, and 1 grade 1 nephrolithiasis event in a participant with a prior history of asymptomatic nephrolithiasis was considered unrelated to the study drug.

**Table 6:** Adverse events through Week 104 among palopegteriparatide-treated participants in the PaTHway trial.

**Treatment emergent adverse events (TEAEs) *n* (%)**	**All participants** ^**a**^ **(*n* = 80)**	**Baseline eGFR <60 mL/min/1.73 m** ^**2**^ **(*n* = 23)**	**Baseline eGFR ≥60 mL/min/1.73 m** ^**2**^ **(*n* = 57)**
Any TEAE	75 (93.8)	22 (95.7)	53 (93.0)
Serious TEAE	14 (17.5)	6 (26.1)	8 (14.0)
TEAE severity^b^			
Grade 1	36 (45.0)	9 (39.1)	27 (47.4)
Grade 2	29 (36.3)	10 (43.5)	19 (33.3)
Grade 3	9 (11.3)	3 (13.0)	6 (10.5)
Grade 4	1 (1.3)	0	1 (1.8)
Related TEAE^c^	44 (55.0)	13 (56.5)	31 (54.4)
Serious related TEAE^d^	2 (2.5)	1 (4.3)	1 (1.8)
TEAE related to hyper- or hypocalcemia leading to ER/urgent care visit and/or hospitalization	6 (7.5)	4 (17.4)	2 (3.5)
TEAE leading to discontinuation of study drug	2 (2.5)	0	2 (3.5)
TEAE leading to death^e^	1 (1.3)	0	1 (1.8)

a Includes TEAEs occurring on or after the first dose of palopegteriparatide in the Safety Analysis Population (participants who received ≥1 dose of palopegteriparatide): 104 wk of exposure for the palopegteriparatide/palopegteriparatide group (*n* = 61) and 78 wk of exposure for the placebo/palopegteriparatide group (*n* = 19).

b Participants are displayed for the highest severity category only.

c Treatment-related adverse events occurring at a rate ≥5% were: injection site reaction (26.3%), hypercalcemia (13.8%), nausea (8.8%), hypocalcemia (7.5%), headache (7.5%), and postural orthostatic tachycardia syndrome (5.0%); no cases of nephrocalcinosis were reported through Week 104, and 1 case of grade 1 nephrolithiasis in a participant with prior medical history of nephrolithiasis was deemed to be unrelated to study drug.

d Hypercalcemia (*n* = 2).

e One participant had a TEAE (fatal cardiac arrest unrelated to study drug) leading to discontinuation of the study drug and death during blinded treatment and is counted in both categories.

## Discussion

Previous reports of eGFR reductions in patients with hypoparathyroidism who are managed with conventional therapy indicate that these patients may experience deterioration in renal function beyond expected age-related changes. This post hoc analysis of the phase 3 PaTHway trial through Week 104 indicates that PTH replacement therapy with palopegteriparatide preserves and may improve renal function in adults with chronic hypoparathyroidism. Concurrent with high efficacy response rates, participants receiving palopegteriparatide showed an improvement in eGFR of 9.0 (10.3) mL/min/1.73 m^2^ from baseline (*p* < .0001) at Week 104, demonstrating preservation of the mean eGFR increase initially reported after 52 wk of treatment. Consistent with results at Week 52, participants randomized to palopegteriparatide demonstrated consistent proportions of participants achieving clinically meaningful eGFR increases of ≥5 and ≥10 mL/min/1.73 m^2^ at Week 104.^1^ Similar mean eGFR increases and eGFR response rates were also observed for participants switched to palopegteriparatide therapy from placebo, demonstrating the reproducibility of the observed changes in renal function. At Week 104, improvements in kidney function were observed in both eGFR subgroups, with numerically more participants with baseline eGFR <60 mL/min/1.73 m^2^ achieving categorical thresholds of eGFR improvement (≥5 and ≥10 mL/min/1.73 m^2^). Importantly, the proportion of participants with an eGFR <60 mL/min/1.73 m^2^ decreased from 28.0% (23/82) at baseline to 9.2% (7/76) at Week 104.

Previous reports from the PaTHway trial demonstrated that treatment with palopegteriparatide significantly improved mean eGFR values by Week 26 for participants with preserved renal function at baseline as well as those with decreased renal function at baseline.^1^ The increase in eGFR during the first 26 wk of palopegteriparatide treatment may be driven by several factors associated with restoration of physiologic PTHR activation, including normalization of renal hemodynamics and renal calcium handling. The observed weak but statistically significant correlations suggest that changes in eGFR are multifactorial, with sparing of conventional therapy as a component. Importantly, maintenance of eGFR increase through Week 104 suggests that these eGFR increases may be the result of beneficial treatment effects, rather than potentially deleterious side effects such as glomerular hyperfiltration or increased intraglomerular filtration pressures.^26^ However, additional longitudinal observation of eGFR in this population is required to confirm the sustained benefit of palopegteriparatide on renal function in hypoparathyroidism.

Regardless of etiology, the development of CKD and transition to kidney failure are, by definition, preceded by reductions in eGFR, with steeper negative eGFR slopes associated with accelerated decline in renal function.^27^ Assessment of eGFR slope may be of value in rare disorders, such as hypoparathyroidism, where evaluation of renal treatment effects based on hard clinical endpoints would require observation of large cohorts over several yr.^28^

In patients with hypoparathyroidism managed with conventional therapy, several reports have noted significantly accelerated eGFR declines compared to unaffected cohorts, with annualized changes in eGFR ranging from −0.7 to −1.8 mL/min/1.73 m^2^/yr.^3^^,^^10^^,^^19^^,^^20^ Treatment with short-lived PTH (rhPTH (1-84), Natpara) has been shown to mitigate the decline in eGFR in patients with chronic hypoparathyroidism for up to 5 yr compared with a historical hypoparathyroidism cohort treated with conventional therapy.^10^ For participants initially randomized to palopegteriparatide, mean eGFR slope from Week 52 to 104 was −0.29 mL/min/1.73 m^2^/yr, which is numerically lower than the expected age-related declines in renal function during normal aging.^29–31^ Given the well-defined renal risks associated with conventional therapy, PTH replacement with palopegteriparatide in patients with hypoparathyroidism may provide critical long-term preservation of renal function as nearly all treated participants achieved independence from active vitamin D and elemental calcium.^32^ As such, the maintenance of eGFR through Week 104 of the PaTHway trial further supports the renoprotective effects of physiologic PTH replacement in adults with hypoparathyroidism.

Limitations of this post hoc analysis include lack of a control or comparator group beyond Week 26, limited demographic diversity, trial exclusion of participants with an eGFR <30 mL/min/1.73 m^2^, and small sample sizes for other subgroups of interest. It is also difficult to draw definitive conclusions regarding the clinical benefit of observed eGFR changes as the precise mechanism for these findings is not yet known; future analyses are planned to explore the nature of these changes. A further limitation is that eGFR was calculated using the MDRD equation, as prespecified in the study protocol. Contemporary guidance recommends the race-free 2021 CKD-EPI equation, which is more accurate at higher GFR and avoids systematic bias^33^; future analyses using CKD-EPI would provide greater precision in assessing renal outcomes. An independent assessment of changes in eGFR using the CKD-EPI equation yielded a similar magnitude of improvement to that observed with the MDRD equation. Regardless, the findings from the PaTHway trial are based on changes in serum creatinine and derivation of eGFR using creatinine-based equations. Serum creatinine levels reflect both changes in glomerular filtration of serum creatinine as well as fluctuations in serum creatinine not due to glomerular filtration (eg, creatinine tubular secretion, extrarenal creatinine clearance, muscle mass, and diet). Moreover, the interpretation of eGFR changes may be confounded by discontinuation of calcitriol, which may lower serum creatinine independent of true changes to GFR.^34^ Future investigation is required to confirm these results using additional analytes to derive eGFR (ie, cystatin C) and by direct measurement of GFR.

In summary, the data from the phase 3 PaTHway trial confirm the efficacy and safety of palopegteriparatide in adults with chronic hypoparathyroidism and demonstrate that early improvements in eGFR over the first 26 wk of palopegteriparatide treatment are maintained through Week 104. These data, together with the positive effects on quality of life and on maintenance of stable serum calcium and phosphate values underscore the generalized benefit of this form of PTH replacement therapy for those suffering from chronic hypoparathyroidism and its biochemical abnormalities and clinical manifestations.

## Supplementary Material

PaTHway_2-Year_Renal_Analysis_Supplementary_Material_zjag085

## Data Availability

The datasets generated during and/or analyzed during the current study are not publicly available but are available from the corresponding author upon reasonable request.

## Previous publication

Parts of these results were presented previously in abstract form at the European Society of Endocrinology (ECE) annual meeting in Stockholm, Sweden, May 11-14, 2024 and the Endocrine Society (ENDO) annual meeting in San Francisco, CA, July 12-15, 2025.
