# Supplementary material for: Sustained improvement in renal function with palopegteriparatide in adults with chronic hypoparathyroidism: 2-year results from the phase 3 PaTHway-trial
Source: J Bone Miner Res. 2026 May 21;41(9):922–33. doi: 10.1093/jbmr/zjag085 (PMC13525197; doi:10.1093/jbmr/zjag085)
Supplement: Supplementary file 1 [file pathway_2-year_renal_analysis_supplementary_material_zjag085.docx]

## Supplemental Table 1: Change in eGFR by treatment group and baseline renal function

|  | All Participants | | Baseline eGFR < 60 mL/min/1.73 m^2^ | | Baseline eGFR ≥ 60 mL/min/1.73 m^2^ | |
| --- | --- | --- | --- | --- | --- | --- |
|  | Palopegteriparatide  (*n* = 61) | Placebo/ Palopegteriparatide (*n* = 21) | Palopegteriparatide  (*n* = 19) | Placebo/ Palopegteriparatide (*n* = 4) | Palopegteriparatide  (*n* = 42) | Placebo/ Palopegteriparatide (*n* = 17) |
| Change from baseline in eGFR at Week 26 (mL/min/1.73 m^2^)^a^ | | | | | | |
| *n* | 60 | 19 | 19 | 4 | 41 | 15 |
| Mean (SD) | 7.9 (10.4) | -1.9 (8.6) | 11.4 (10.7) | 0.1 (6.0) | 6.3 (10.0) | -2.4 (9.3) |
| 95% CI^b^ | 5.2, 10.6 | -6.1, 2.2 | 6.2, 16.5 | -9.5, 9.6 | 3.1, 9.5 | -7.6, 2.7 |
| *p* value^c^ | < 0.0001 | 0.3468 | 0.0002 | 0.9877 | 0.0002 | 0.3280 |
| Change from baseline in eGFR at Week 52 (mL/min/1.73 m^2^)^d^ | | | | | | |
| *n* | 59 | 19 | 19 | 4 | 40 | 15 |
| Mean (SD) | 9.3 (11.7) | 7.6 (8.7) | 11.5 (11.3) | 11.7 (2.2) | 8.2 (11.9) | 6.5 (9.5) |
| 95% CI^b^ | 6.2, 12.3 | 3.4, 11.8 | 6.0, 16.9 | 8.1, 15.2 | 4.4, 12.1 | 1.2, 11.7 |
| *p* value^c^ | < 0.0001 | 0.0014 | 0.0003 | 0.0018 | < 0.0001 | 0.0199 |
| Change from baseline in eGFR at Week 104 (mL/min/1.73 m^2^)^e^ | | | | | | |
| *n* | 58 | 18 | 18 | 4 | 40 | 14 |
| Mean (SD) | 8.9 (10.6) | 9.4 (9.4) | 13.4 (10.9) | 15.6 (4.6) | 6.9 (10.0) | 7.6 (9.7) |
| 95% CI^b^ | 6.4, 11.1 | 6.3, 14.8 | 9.3, 17.7 | 6.0, 24.8 | 3.9, 9.5 | 4.1, 13.5 |
| *p* value^c^ | < 0.0001 | 0.0067 | < 0.0001 | 0.0121 | < 0.0001 | 0.0006 |

eGFR, estimated glomerular filtration rate; CI, confidence interval; SD, standard deviation. ^a^Reflects 26 weeks of palopegteriparatide or placebo treatment. ^b^Calculated from an ANCOVA model with change from baseline in eGFR as a dependent variable, treatment, baseline eGFR group, and sex as fixed effects, and baseline eGFR and age as covariates. ^c^Derived from a paired *t*-test, testing if the change is > 0. ^d^All participants received palopegteriparatide from week 26 to 52, reflecting 52 weeks of palopegteriparatide treatment for palopegteriparatide participants and 26 weeks of palopegteriparatide treatment for placebo/palopegteriparatide participants. ^e^All participants received palopegteriparatide from week 26 to 104, reflecting 104 weeks of palopegteriparatide treatment for palopegteriparatide participants and 78 weeks of palopegteriparatide treatment for placebo/palopegteriparatide participants.

## Suppl. Fig. 1: Change from baseline in eGFR in the PaTHway trial through Week 104 by weeks on palopegteriparatide treatment

## Suppl. Fig. 2: eGFR through Week 104 in the PaTHway trial (all participants)
